# Spondylodiscitis Following Perforated Acute Appendicitis: A Case Report

**DOI:** 10.7759/cureus.62816

**Published:** 2024-06-21

**Authors:** Polina Angelova, Atanas Davarski, Ivo Kehayov, Borislav Kitov

**Affiliations:** 1 Department of Neurosurgery, Medical University of Plovdiv, Plovdiv, BGR; 2 Department of Neurosurgery, Sv. Georgi University Hospital, Plovdiv, BGR

**Keywords:** spinal infection, surgical treatment, spine, acute appendicitis, spondylodiscitis

## Abstract

Spondylodiscitis is a multifactorial disease of significant medical and socioeconomic importance, the treatment of which presents a challenge to clinicians and surgeons. Acute appendicitis is a common inflammatory disease in children, with postoperative complications occurring in up to 55% of cases. We present the case of a 15-year-old male with symptoms of severe back pain and fever two months following surgery for perforated appendicitis. The computed tomography (CT) revealed spondylodiscitis of T12-L1 spinal level. Discectomy and posterior pedicle-screw fixation were performed, followed by antibiotic treatment resulted in the resolution of preoperative symptoms. To the best of our knowledge, this is the third case of spondylodiscitis after perforated acute appendicitis in literature. Timely diagnosis and treatment in cases of spondylodiscitis are prerequisites for lowering the rate of permanent neurological deficits in these patients.

## Introduction

Acute appendicitis is one of the most common causes of emergency abdominal surgery worldwide, with an incidence of 100 per 100,000 people per year. In approximately 29% of cases, appendicitis is perforated [1]. In children, the incidence of perforated appendicitis is about 30%, as it can be much higher in younger children [2]. Morbidity and mortality are significantly higher in cases of perforated appendicitis (16%) in contrast with non-perforated appendicitis (5.6%) [1]. According to Ponsky et al., the risk of abdominal abscess, wound infection, or postoperative ileus is 39% in perforated and 8% in non-perforated appendicitis [3]. Other possible complications, such as enterocutaneous fistula and small bowel obstruction, are also reported [4]. The occurrence of spontaneous spondylodiscitis following surgery for perforated appendicitis in children is an extremely rare complication, and to the best of our knowledge, only two similar cases have been reported in the literature [1,5]. We present a rare case of spontaneous spondylodiscitis following perforated acute appendicitis.

## Case presentation

We present the case of a 15-year-old patient with no previous medical history. The patient underwent surgery for purulent perforated appendicitis with local peritonitis. Following the appendectomy, the patient received antibiotic treatment with Amikacin, Meropenem, and Metronidazole for seven days. Two months later he presented with a fever (≥38°C) and severe back pain, which was increasing when standing. The blood pressure, pulse rate, and respiratory rate were normal for age. The symptoms were not relieved by conservative treatment with non-steroid anti-inflammatory drugs. Upon hospital admission, the neurological examination revealed severe back pain. No motor or sensory deficit was established.

**Figure 1:**
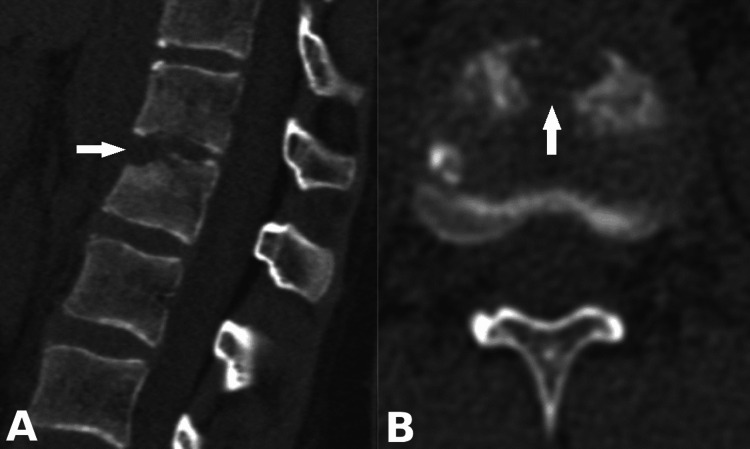
(A) Sagittal and (B) axial preoperative CT images showing the destruction of the lower endplate of T12 and upper endplate of L1 vertebral bodies (arrows)

Laboratory tests revealed leukocytosis (15.25 x 10^9 g/l), elevated erythrocyte sedimentation rate (47 mm/h), and C-reactive protein (57 mg/l). The thoracolumbar section CT presented the destruction of the lower endplate of T12 and the upper endplate of L1 - a neuroimaging characteristic of spondylodiscitis at the T12-L1 level (Figure 1).

**Figure 2:**
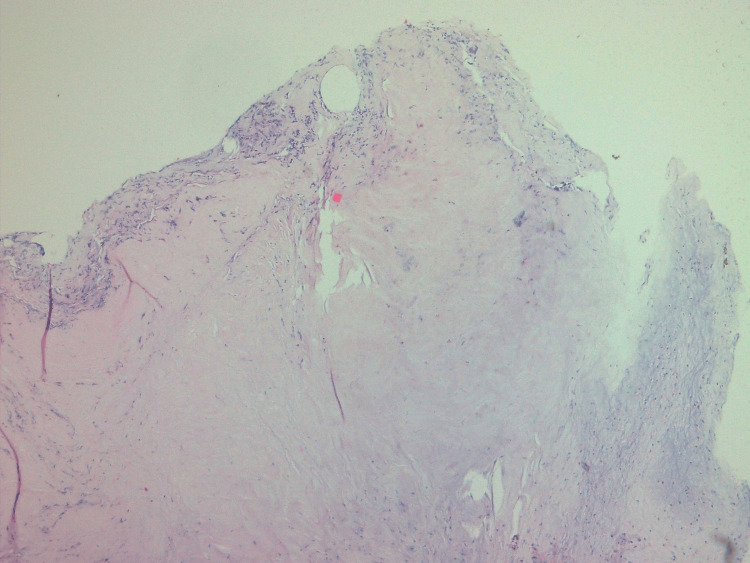
Histological examination demonstrating degenerative intravertebral disc with infectious foci (HE × 40)

**Figure 3:**
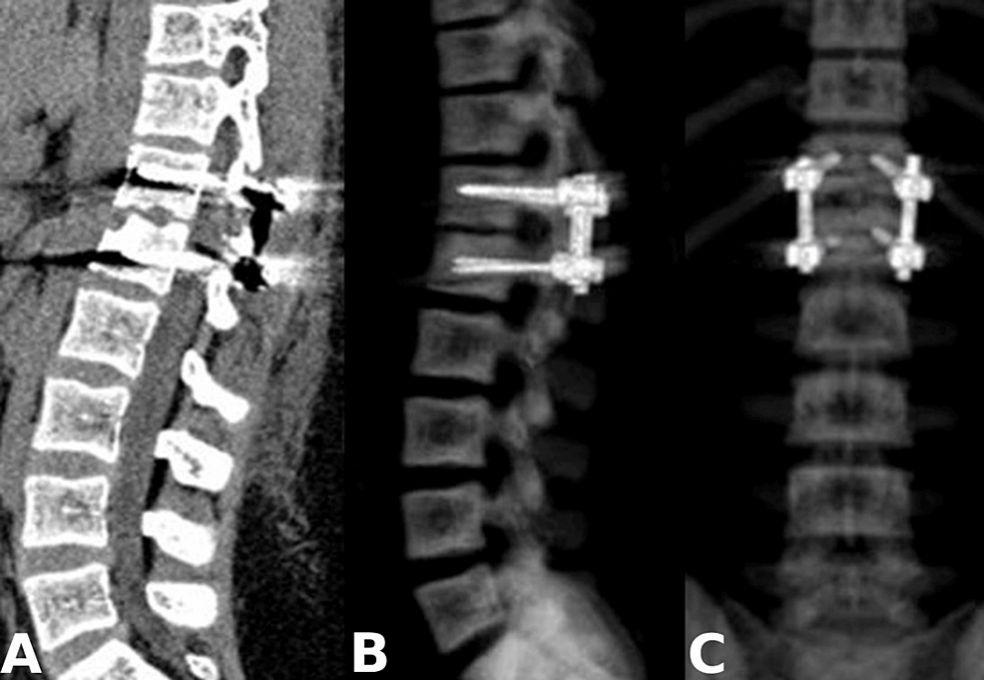
Postoperative CT. (А) Sagittal reconstruction and (B and C) sagittal and coronal 3D-reconstruction showing posterior transpedicular fixation of T12 and L1 vertebral bodies

The neuroimaging data presented involvement of the anterior and middle spinal columns, which suggests future spinal instability. Therefore, the patient was operated on via right interlaminotomy and discectomy of the T12-L1 level. The intervertebral disc showed signs of chronic inflammation, consisting of fragments and bone sequestrations. Given the marked destruction of the T12 and L1 vertebral bodies and aiming to prevent future spinal instability, a short-segment posterior pedicle-screw fixation was subsequently performed (Figure 2). Short-segment fixation was performed considering the age of the patient and possible growth inhibition. The microbiological cultures were sterile. The histological examination revealed a degenerative intravertebral disc with infectious foci (Figure 3).

Following surgery, antibiotic treatment with Ceftriaxone and Vancomycin was administered for two weeks.

The postoperative period was uneventful, with complete resolution of the preoperative symptoms.

## Discussion

Spinal infections include discitis, vertebral osteomyelitis, abscess, or spondylodiscitis. In cases of spondylodiscitis, the infection affects both the intervertebral disc and the adjacent vertebral bodies [6]. Spondylodiscitis represents 3-5% of all cases of osteomyelitis with annual incidence from 0.4 to 2.4 cases per 100,000 and a mortality rate reaching 11% [7].

Spondylodiscitis is rarely diagnosed in children with an annual incidence of 0.3 cases per 100,000, accounting for 3% of childhood osteoarticular infections [8]. The peak of the disease is observed at the age of 10-15 years, with a male predominance (m:f = 1.5-2:1) [1].

Pathogens can infect the spine in three ways: a) hematogenous dissemination (arterial or via Batson's venous plexus), b) direct external inoculation, and c) per continuitatem from adjacent affected tissues. Spondylodiscitis most often occurs through hematogenous spread from a distant infectious focus in the body [9]. In children, the arteries of the vertebral bodies have significant anastomoses with the arterial vessels penetrating the intervertebral disc [10]. With age, the disc gradually becomes avascular, and bacterial infection is prone to affect directly the vertebral body [11]. In our case, the infection started at the endplates of the vertebral bodies.

In cases of neurological deficits, spinal instability, bone destruction, or abscess formation, surgical treatment is required. Possible operative interventions for spondylodiscitis in the thoracolumbar region include anterior and posterior surgical approaches. Anterior access provides effective and efficient focal debridement, restoration of spinal stability, bone fusion, neural decompression, and deformity correction [12]. However, it is associated with greater surgical trauma and higher complication rates, such as including vascular and visceral injuries [13]. In recent years, posterior access has been increasingly used in spondylodiscitis in the thoracolumbar segment, including one-stage posterior debridement and instrumentation. The advantages of posterior access in thoracolumbar surgery include familiarity with the approach and widespread use of posterior pedicle screw placement among spinal surgeons [14]. Short and long spinal fusion techniques are almost equally used in cases of spondylodiscitis as long fixation is used more frequently in thoracolumbal junction [15]. In our case, short-segment fixation was performed considering the age of the patient and the possibility of growth inhibition.

There are discussions regarding the risk of implant infection following fusion surgery in cases of spondylodiscitis. Spinal instrumentation is a risk factor for contamination. However, the rate of re-operations due to infection relapse is higher in cases of decompression alone [16].

According to the literature, the most common pathogens causing hematogenous spondylodiscitis in children are *Staphylococcus aureus*, *Streptococcus pyogenes*, and *Streptococcus pneumoniae*. The incidence of sterile microbiological cultures varies from 21% to 41.7% [17,18]. In our case, the difficulty in isolating pathogens is due to the antibiotic therapy carried out following appendicitis surgery. In similar cases, other methods of detecting non-culturable bacteria, such as PCR, should be performed [19]. 

In the presented case, spondylodiscitis most likely occurred due to transferring the pyogenic microorganisms into the retroperitoneum during the laparotomy, as well as retrograde venous reflux from the pelvis to the paravertebral plexus due to the lack of valves in the draining spinal veins [20]. We present a comparison of our case and two similar cases that have been reported in the literature (Table 1).

**Table 1:** Comparison of three cases of appendicitis followed by spondylodiscitis according to literature

Case	Age	Gender	Location	Type of surgery	Microbiological finding	Conservative treatment
Çelik and Can [5]	24 yo	Female	L5-S1	None	None	Intravenous Ciprofloxacin and Ampicillin sulbactam followed by oral Ciprofloxacin and Sultamicillin
AlTarayra et al. [1]	14 yo	Female	L3-L4	None	Pseudomonas aeruginosa	Intravenous Vancomycin and Ceftazidime followed by Meropenem and Vancomycin
Our case	15 yo	Male	T12-L1	T12-L1 spinal fusion	Sterile cultures	Intravenous Ceftriaxone and Vancomycin

## Conclusions

Spondylodiscitis remains a severe and disabling spinal disease with a high mortality rate despite advances in surgical and antibiotic treatment. This condition should be considered in cases of children with back pain, radiculopathy, or myelopathy associated with fever and elevated inflammatory markers following acute appendicitis surgery. Prompt diagnosis and treatment are crucial for the outcome and the timely recovery of patients.
